# Functional Analysis of *Leishmania* Cyclopropane Fatty Acid Synthetase

**DOI:** 10.1371/journal.pone.0051300

**Published:** 2012-12-10

**Authors:** Samuel O. Oyola, Krystal J. Evans, Terry K. Smith, Barbara A. Smith, James D. Hilley, Jeremy C. Mottram, Paul M. Kaye, Deborah F. Smith

**Affiliations:** 1 Centre for Immunology and Infection, Department of Biology/Hull York Medical School, University of York, York, United Kingdom; 2 Biomedical Sciences Research Complex, University of St. Andrews, Fife, United Kingdom; 3 Wellcome Trust Centre for Molecular Parasitology, Institute of Infection, Immunity and Inflammation, College of Medical, Veterinary and Life Sciences, University of Glasgow, Glasgow, United Kingdom; Louisiana State University, United States of America

## Abstract

The single gene encoding cyclopropane fatty acid synthetase (CFAS) is present in *Leishmania infantum*, *L. mexicana* and *L. braziliensis* but absent from *L. major*, a causative agent of cutaneous leishmaniasis. In *L. infantum*, usually causative agent of visceral leishmaniasis, the CFAS gene is transcribed in both insect (extracellular) and host (intracellular) stages of the parasite life cycle. Tagged CFAS protein is stably detected in intracellular *L. infantum* but only during the early log phase of extracellular growth, when it shows partial localisation to the endoplasmic reticulum. Lipid analyses of *L. infantum* wild type, CFAS null and complemented parasites detect a low abundance CFAS-dependent C19Δ fatty acid, characteristic of a cyclopropanated species, in wild type and add-back cells. Sub-cellular fractionation studies locate the C19Δ fatty acid to both ER and plasma membrane-enriched fractions. This fatty acid is not detectable in wild type *L. major*, although expression of the *L. infantum* CFAS gene in *L. major* generates cyclopropanated fatty acids, indicating that the substrate for this modification is present in *L. major*, despite the absence of the modifying enzyme. Loss of the *L. infantum* CFAS gene does not affect extracellular parasite growth, phagocytosis or early survival in macrophages. However, while endocytosis is also unaffected in the extracellular CFAS nulls, membrane transporter activity is defective and the null parasites are more resistant to oxidative stress. Following infection *in vivo, L. infantum* CFAS nulls exhibit lower parasite burdens in both the liver and spleen of susceptible hosts but it has not been possible to complement this phenotype, suggesting that loss of C19Δ fatty acid may lead to irreversible changes in cell physiology that cannot be rescued by re-expression. Aberrant cyclopropanation in *L. major* decreases parasite virulence but does not influence parasite tissue tropism.

## Introduction

*Leishmania* are obligate intracellular protozoan parasites that infect humans and other mammalian species causing broad spectrum diseases termed the leishmaniases. Parasites are transmitted as extracellular flagellated forms (metacyclic promastigotes) by female sandflies during blood feeding [1]. Once in the host, the metacyclic promastigotes are phagocytosed by host cells (including neutrophils and macrophages) and differentiate into replicative amastigotes within intracellular phagolysosomal compartments. Maintenance of parasites at dermal sites or subsequent dispersal to internal tissues contributes to disease progression, resulting in the distinct pathologies associated with cutaneous (CL), mucocutaneous (MCL), diffuse cutaneous (DCL) and visceral leishmaniases (VL) [2], [3]. These diseases are often associated with particular parasite species: *L. infantum* and *L. major* usually causing VL and CL respectively, while *L. braziliensis* is a major causative agent of MCL. The immune response to infection in the host also has a dominant role in determining clinical outcome (reviewed in [4]).

The genome sequences of *L. major, L. infantum, L. braziliensis, L. donovani* and *L. mexicana* have been published [5], [6], [7], [8]. Comparative analysis of these five published reference genomes has identified only a few species-specific genes that could be implicated in contributing to parasite tissue tropism and disease pathogenesis in the host, following infection with different *Leishmania* species. Most of these genes code for proteins that share low identity with functionally characterised molecules from other organisms [5], [7], [8], [9]. One exception is an orthologue of the metabolic enzyme, cyclopropane fatty acid synthetase (CFAS), which is present in the *L. infantum*, *L. donovani*, *L. braziliensis* and *L. mexicana* genomes but absent from *L. major* and other kinetoplastids including *Trypanosoma* species [5]. A CFAS-like sequence (Cf_Contig1069, WUSTL, P value 0.00041) has been found in the recently sequenced genome of *Crithidia fasciculate* however. Phylogenetic analysis suggests that the *Leishmania* genus acquired the CFAS gene by horizontal transfer (probably from bacteria) with secondary loss from *L. major* [5].

CFAS enzymes catalyse the cyclopropanation of unsaturated fatty acids, a reaction which, in bacteria, involves the transfer of a methylene group from a *S*-adenosyl-*L*-methionine (SAM) donor to a carbon-carbon double bond within a fatty acyl chain [10]. Although the position of the *cis* double bond on the acyl chain is variable in *Escherichia coli, Mycobacterium tuberculosis* produces several site-specific cyclopropane synthetases that modify mycolic acids [11]. Cyclopropanation of the *M. tuberculosis* cell envelope mycolates has been shown to play a role in the modulation of host innate immune responses during infection, a response associated with pathogen persistence in the host [12]. A physiological role for cyclopropanation has not been fully elucidated in other bacterial species, however, although CFAS-catalysed membrane modifications have been associated with stress responses to changes in pH, temperature or salinity of the local environment in *E. coli* [13]. Most recently, CFAS mutants of the probiotic bacterium, *Lactobacillus reuteri*, have been shown to be defective in inhibiting the TNF (tumor necrosis factor) immunomodulatory activity that characterises certain human-derived strains but this is an indirect effect, postulated to be due to a decrease in bacterial membrane fluidity [14].

Here we describe functional characterisation of the *L. infantum* cyclopropane fatty acid synthetase, which is expressed in both promastigote (extracellular) and amastigote (intracellular) parasite forms. The membrane-associated enzyme is required for fatty acid modification in *L. infantum*, generating cyclopropanated fatty acids. Interestingly, expression of a CFAS transgene in *L. major* parasites which normally lack the single copy CFAS gene generates cyclopropanated fatty acids, suggesting that the substrate for this modification may be common to all *Leishmania* species. Loss of the CFAS gene in *L. infantum* does not affect promastigote growth or phagocytosis by macrophages *in vitro* but does appear to influence membrane transport and resistance to oxidative stress. Animal studies indicate that CFAS loss can also compromise parasite survival *in vivo* but rescue of this phenotype has not been achieved, despite rescue of the biochemical phenotype by complementation in the infecting parasites.

## Results

### Expression and Localisation of Cyclopropane Fatty Acid Synthetase in *Leishmania infantum*

Comparative analysis of the *Leishmania* reference genomes identified a gene orthologue encoding CFAS that is present in *L. infantum*, *L. braziliensis* and *L. mexicana* but absent from *L. major* [5], [7]. This gene (LinJ.08.0560, located on chromosome 8 of *L. infantum)* encodes a 55 kDa protein that shares 48% amino acid similarity with CFAS-encoding genes of *Mycobacterium tuberculosis* and *Escherichia coli*. Primary sequence alignment [15] identified a structurally conserved S-adenosyl-*L*-methionine (SAM) binding domain (residues 265–363) together with other conserved amino acids characteristic of this class of enzymes (Figure S1).

**Figure 1:**
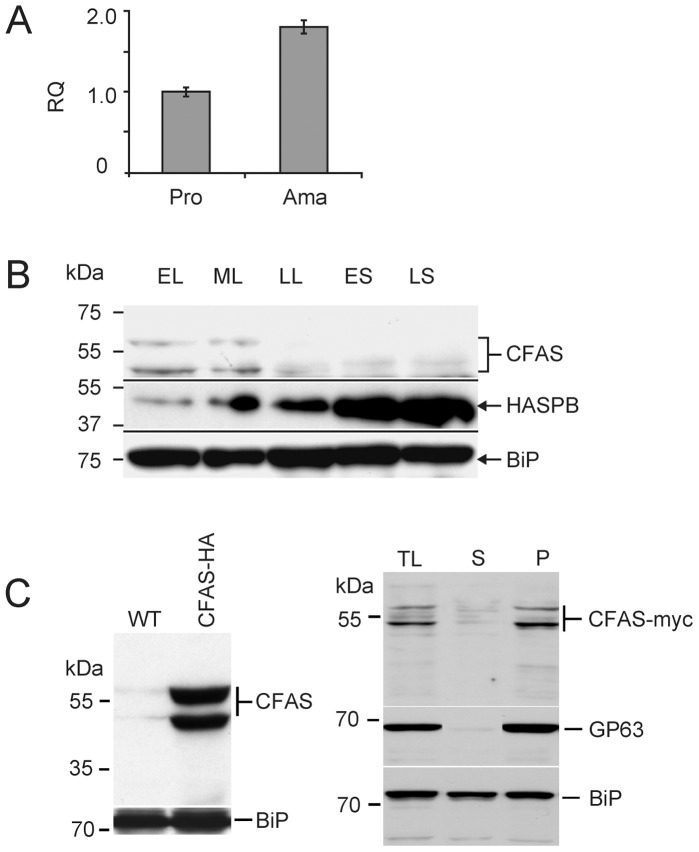
CFAS expression in *L. infantum*. (A) Quantitative analysis (RT-qPCR) of CFAS RNA expression in mid-log promastigotes (Pro) and amastigotes (Ama). RQ, relative quantity with reference to EF1α control; error bars represent standard error of mean. (B) Immunoblotting analysis of *L. infantum* promastigotes expressing C-terminal myc-tagged CFAS (CFAS-myc) harvested in early (EL), mid (ML) and late (LL) log phase growth and in early (ES) and late (LS) stationary phase growth. CFAS-myc is detected by anti-myc; the metacyclic protein HASPB is a marker for *in vitro* differentiation; the ER marker BiP is constitutively expressed during the *Leishmania* growth cycle. (C) Left: immunoblotting of wild type (WT) and early log phase *L. infantum* CFAS-HA promastigote total lysates. Right: fractionation of CFAS-HA total lysate (TL) into cytosolic (soluble, S) or membrane (pellet, P) fractions prior to immunoblotting. GP63, membrane-specific marker; BiP, loading control.

Using RT-qPCR to analyse CFAS mRNA levels during the *L. infantum* life cycle, CFAS transcripts were detected in both promastigotes and amastigotes, indicative of constitutive expression in both extracellular and intracellular stages of the parasite life cycle. Quantitative analysis showed a two-fold higher mRNA abundance in tissue-derived amastigotes compared to promastigotes (Figure 1A). Given the absence of transcriptional regulation as a general mechanism for the control of gene expression in kinetoplastid species including *Leishmania* [16], these data are consistent with increased stabilisation of CFAS transcripts in intracellular amastigotes. To investigate expression of CFAS protein, a C-terminally myc-tagged CFAS was transfected into *L. infantum* promastigotes and protein expression monitored during the extracellular growth phase by immunoblotting (Figure 1B). This analysis identified two proteins, of ∼55 kDa (the predicted size for CFAS) and ∼53 kDa, which were detectable only during the early and mid-logarithmic stage of growth. In the same parasites, increased expression of the metacyclic marker protein, HASPB, was detected in stationary phase as shown previously in *L. major* and *L. donovani* [17], [18], [19], [20], while the constitutive marker, BiP, was expressed throughout the growth cycle [21]. The 55 kDa CFAS-myc protein is more unstable than the smaller protein, degrading within hours if kept at 4°C or after 7 days of storage at −20°C (data not shown). To investigate this further, an alternative C-terminally HA-tagged CFAS mutant line was generated and analysis of mid log promastigote lysates again showed expression of two HA-tagged CFAS proteins (Figure 1C, left-hand panel). Sub-cellular fractionation following lysis of these parasites, using differential centrifugation to separate cytosolic and membrane-containing fractions prior to immunoblotting, detected both CFAS-HA proteins predominantly in the membrane fraction, suggesting that CFAS is membrane-associated in *Leishmania* (Figure 1C, right-hand panel). As expected, the surface GPI-anchored *Leishmania* protein, GP63 [22], [23] fractionated as an exclusively membrane protein while BiP separated between both cytosolic and membrane fractions in this analysis [21]. The two HA-tagged CFAS isoforms detected in this analysis may result from either co- or post-translational modifications (that might explain the membrane-localisation of the CFAS protein) but these are unlikely to involve the protein termini as the same expression pattern was detected using a third, N-terminal HA-tagged CFAS protein (data not shown). Both products are rapidly degraded as the parasites enter late logarithmic and stationery phases of growth (Figure 1B).

**Figure 2:**
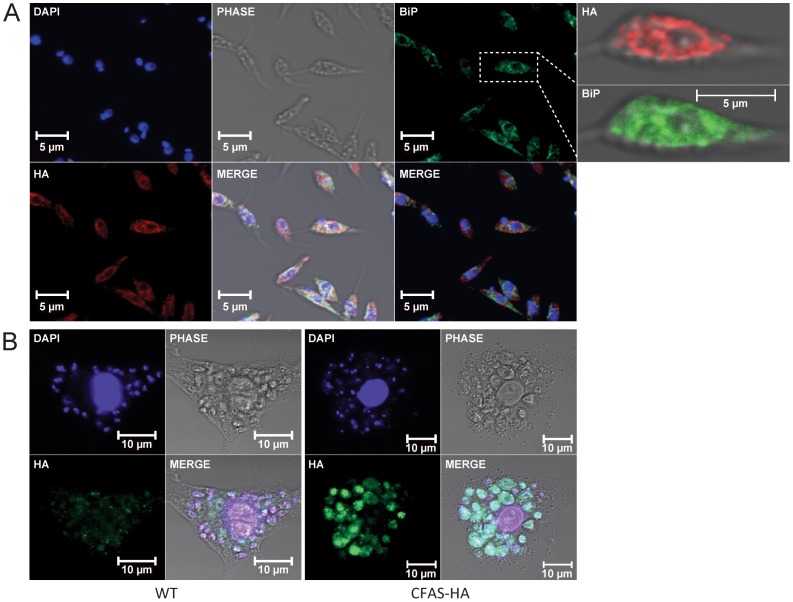
Subcellular localization of CFAS-HA in *L. infantum*. (A) Confocal images of *L. infantum* promastigotes expressing CFAS-HA. Cells were co-stained with anti-HA (red) and anti-BiP (green) and mounted with Vectorshield containing DAPI (blue, DNA). Higher magnification images of a co-stained parasite are shown on the right of the Figure. (B) Confocal images of CFAS-HA expression in intra-macrophage amastigotes at 72 hr post-infection. Macrophages infected with wild type (WT) *L. infantum* (left panel) and *L. infantum* expressing CFAS-HA (right panel). DAPI (blue), macrophage and parasite DNA; anti-HA (green), CFAS-HA.

The *E. coli* CFAS is unstable [24], [25], [26], a property shared by the *L. infantum* CFAS protein when expressed in bacteria, making the production of recombinant protein for biochemical analysis or antibody generation a major challenge. In the absence of an antibody for detection of wild type CFAS protein, subcellular localisation was investigated by immunofluorescence microscopy of promastigotes and amastigotes expressing the C-terminally HA-tagged expression construct (described above). CFAS-HA signal was detected in the cytoplasm but the strongest signal in promastigotes was detected in the perinuclear region and showed some co-localisation with the ER protein BiP [21], suggesting that CFAS is at least partially localised in the ER (Figure 2A). To investigate expression in amastigotes, late stationary phase HA-tagged CFAS mutant promastigotes were used to infect bone marrow-derived macrophages *in vitro*. Infected macrophages were fixed at 72 hr post-infection and expression of CFAS analysed by immunofluorescence. As shown in Figure 2B, strong staining for CFAS-HA fusion protein was detectable at this time point, indicating that stable CFAS protein can be detected in intracellular amastigotes, in keeping with the increased RNA expression levels observed in Fig. 1A.

### Generation of *L. infantum* CFAS Mutant Parasites

**Figure 3:**
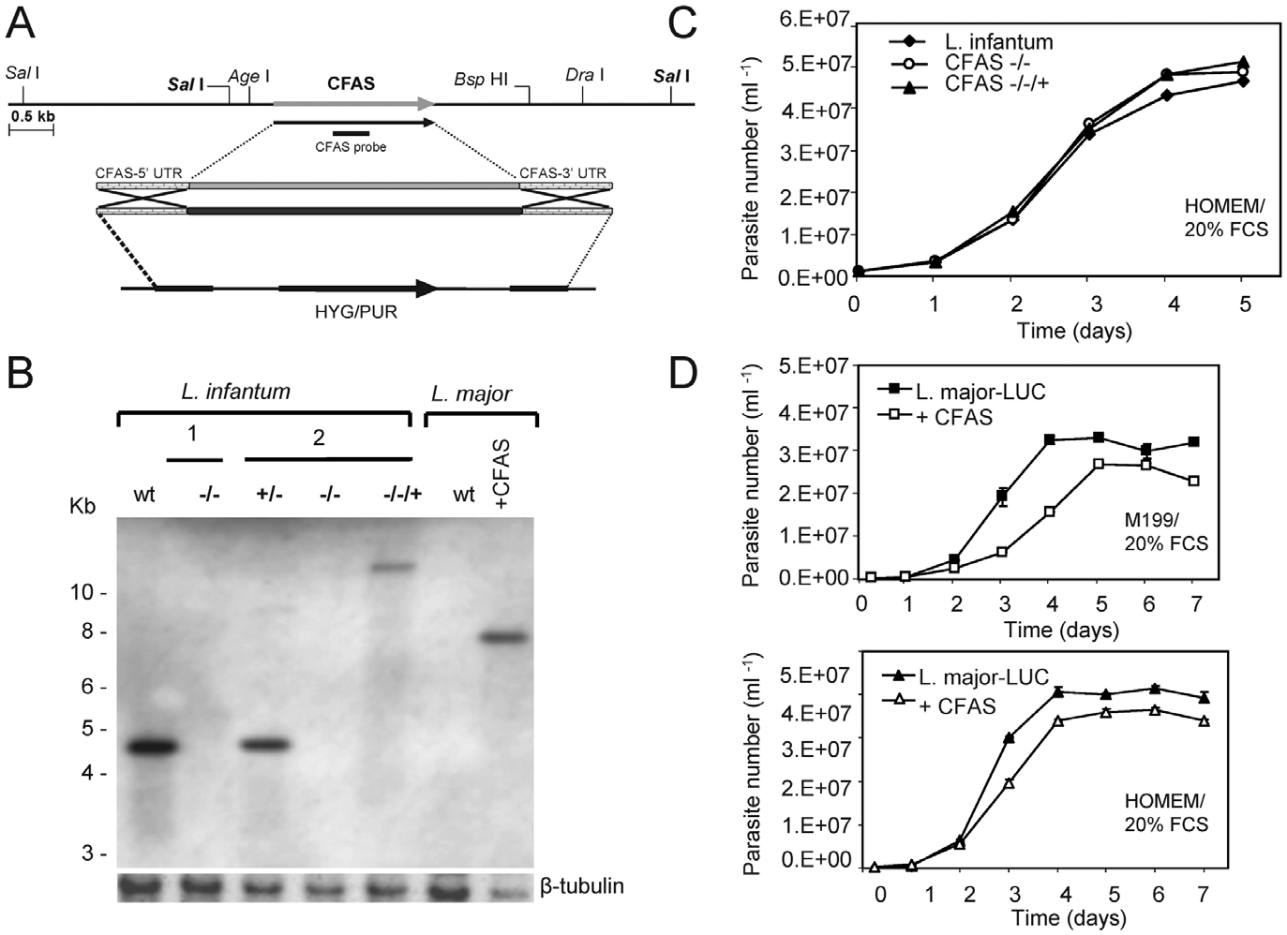
Generation of CFAS mutant parasites and *in vitro* growth analysis. (A) Region of chromosome 8 containing the single *L. infantum CFAS* locus and the constructs used for targeted gene deletion. (B) Representative Southern blot of *L. infantum* and *L. major* wild type (wt) and CFAS mutant DNAs hybridised with a CFAS-specific probe (see A above and Materials and Methods). Two independent *L. infantum* CFAS null clones (1 and 2, −/−) are shown; the single allele deletion prior to generation of null clone 2 (+/−) and a complemented add-back clone from that line (−/−/+, **CLN-C2**, Table 1) are included. One of the clones of *L. major* transgenic for luciferase and CFAS (+CFAS, **CLN-4**, Table 1) is also shown. A labelled β-tubulin specific DNA probe was used as a loading control. (C) *In vitro* growth of *L. infantum* cell lines. *L. infantum* wild type and CFAS null and complemented lines (as analysed in (B)) were grown over 5 days in HOMEM/20% FCS at 26°C and parasites counted as described (Materials and Methods). Mean values derived from triplicate culture populations for each cell line are plotted. (D) *In vitro* growth of *L. major* cell lines. *L. major* wild type containing an integrated luciferase gene (*L. major* LUC) and the same line expressing CFAS (analysed in B) were grown over 7 days in either M199/20% FCS (top) or HOMEM/20% FCS (bottom) and parasites counted as described in (C).

To facilitate functional characterization of CFAS, the single gene was deleted from the genome of *L. infantum* by targeted gene disruption, using constructs that replaced each of the two alleles with either a hygromycin or puromycin cassette (Figure 3A). A number of complemented cell lines containing a single “add-back” gene were also generated. These were designed to express CFAS from either the ribosomal locus ([+pSSU NEO CFAS] constructs) or following integration back into the chromosome of origin ([+CFAS::NEO] constructs) in the null mutant (CFAS−/−, *ΔCFAS*::HYG/*ΔCFAS*::PAC) genetic background (Table 1).

**Table 1:** C19Δ fatty acid content of wild type and CFAS mutant *L. infantum* and *L. major* promastigotes.

Cell line	C19Δ fatty acid (nM/10^9 ^cells)
*L. infantum* +/+	37.6±5
*L. infantum ΔCFAS*::HYG/*ΔCFAS*::PAC	0.0±0
*L. infantum ΔCFAS*::HYG/*ΔCFAS*::PAC [+pSSU NEO CFAS] **CLN-C2**	141.2±17
*L. Infantum ΔCFAS*::HYG/*ΔCFAS*::PAC [+CFAS::NEO] **CLN-D12**	15±8
*L. Infantum ΔCFAS*::HYG/*ΔCFAS*::PAC [+CFAS::NEO] **CLN-E7**	28±0.6
*L. Infantum ΔCFAS*::HYG/*ΔCFAS*::PAC [+CFAS::NEO] **CLN-A6**	9±5
*L. maj*or-LUC +/+	0.0±0
*L. major-*LUC [+pSSU NEO CFAS] **CLN-1**	320.0±21
*L. major-*LUC [+pSSU NEO CFAS] **CLN-2**	212.0±11
*L. major-*LUC [+pSSU NEO CFAS] **CLN-3**	444.0±27
*L. major-*LUC [+pSSU NEO CFAS] **CLN-4**	212.0±14

Derivatised fatty acid extracts from all parasites lines were analysed as described (Materials and Methods); data (mean ± SD, n = 3) are shown from (i) one clone each of wild type (+/+) *L. major*-LUC, +/+ *L. infantum* and CFAS null (*ΔCFAS* ::HYG/*ΔCFAS* ::PAC) *L. infantum;* (ii) four *L. infantum* CFAS null clones complemented by CFAS expression from either the ribosomal *(*[+pSSU NEO CFAS]) or endogenous ([+CFAS::NEO]) locus. Data are also included for four clones of *L. major*-LUC expressing CFAS from the ribosomal locus. All clone names, as used in the text, are in bold.

**Figure 4:**
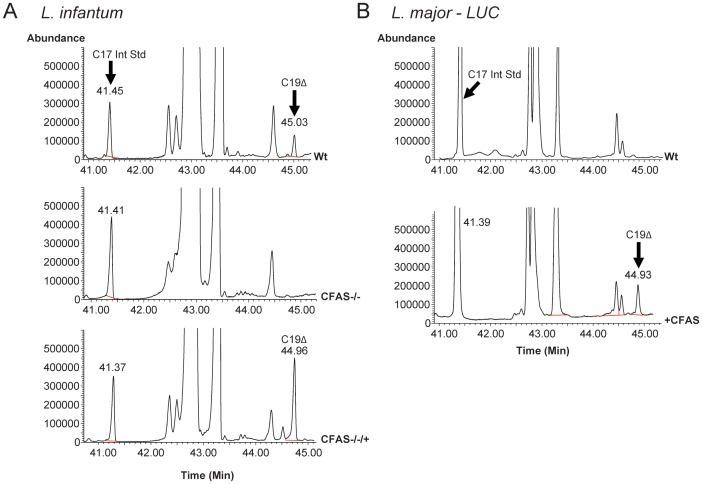
Fatty acid analysis of wild type and CFAS mutant *Leishmania* by gas chromatography–mass spectrometry. (A) Total ion chromatogram of derivatised fatty acid extracts from *L. infantum* wild type (Wt), CFAS null mutant (CFAS−/−) and CFAS complemented (−/−/+; Table 1, **CLN-C2**) cell lines. A spectral peak with a retention time of ∼45 min and corresponding to C19 cyclopropanated fatty acid (C19Δ) is present in wild type and complemented parasites but absent from the CFAS−/− null. (B) Total ion chromatogram of derivatised fatty acid extracts from *L. major* wild type (Wt) and CFAS expressing (+CFAS; Table 1, **CLN-4**) parasites. The spectral peak corresponding to C19Δ, with a retention time of ∼45 min, is absent in *L. major* wild type but present in extracts from *L. major* parasites expressing CFAS. The identity of C19Δ FAME was confirmed by comparison with bacterial C17Δ and C19Δ FAME standards.

As wild type *L. major* parasites lack the CFAS gene, transgenic *L. major* expressing *L. infantum* CFAS were also generated, in order to test whether cyclopropanation could occur when the CFAS gene was present and if so, the functional consequence of aberrant cyclopropanation in this species. Successful creation of the CFAS mutant cell lines was confirmed by Southern blot analysis (an example is shown in Figure 3B) and fatty acid analysis (Figure 4). There were no promastigote growth phenotypes associated with CFAS deletion in *L. infantum* (Figure 3C) and only a minor growth retardation, dependent on culture conditions, following expression of CFAS in *L. major* (Figure 3D).

### Confirmation of CFAS Function Using Mutant Parasites

The catalytic activity of CFAS was confirmed by analysis of the fatty acid content of wild type parasites and the different transgenic lines using gas chromatography-mass spectrometry (GC-MS). As shown in Figure 4A, a spectral peak corresponding to CFAS-modified fatty acid (C19Δ), with a retention time of ∼45 min, was identified in wild type parasites but absent in promastigotes of the *L. infantum* CFAS null line (CFAS−/−, *ΔCFAS*::HYG/*ΔCFAS*::PAC). This fatty acid represents a minor component (approximately 0.2%) of the total promastigote fatty acid content. The CFAS−/− cells showed a complete loss of the C19Δ species without any dramatic affect on the total fatty acid content of these parasites. The cyclopropanated fatty acid was restored upon ectopic expression of the CFAS gene in the CFAS−/− cell lines, indicating that this component is a CFAS-modified product. Quantitation of the C19Δ fatty acid level in the add-back line (**CLN2-C2,** CFAS−/−/+) showed a 3.5 fold increase compared to that present in wild-type *L. infantum* (Table 1).

These analyses confirm that the single CFAS gene is expressed as a functional protein in *L. infantum*. To investigate whether CFAS activity can also produce cyclopropanated fatty acids in *L.major*, mutant lines were generated expressing CFAS following gene integration into the ribosomal locus ([+pSSU NEO CFAS]) and their fatty acid content analysed. No C19Δ fatty acid was detected in wild type *L. major* parasites (Figure 4B) but a peak corresponding to C19Δ fatty acid, with a retention time of 45 min, was detected in the CFAS mutant cell line (**CLN-4**, +CFAS). Quantitatively, a six-fold increase in C19Δ fatty acid was found compared to that measured in *L. infantum* wild type cells (Table 1). Thus, these transgenic parasites, together with the *L. infantum* CFAS complemented line CLN-C2 (CFAS−/−/+) are assumed to be over-expressing CFAS protein following random gene integration into the ribosomal locus. To investigate this over-expression phenotype further, a number of other lines were generated and the transgene integration sites within the ribosomal locus mapped using pulsed-field gel electrophoresis and Southern blotting (Figure S2). This analysis demonstrated a correlation between the site of transgene integration relative to the position of the ribosomal promoter and the expression of active protein, as monitored by production of cyclopropanated fatty acid (Table 1). In the two examples shown, the dominant hybridising fragment is smaller in CLN-3 than in CLN- 2, indicative of DNA integration closer to the ribosomal promoter. Other clones in which integration occurred further downstream of the promoter produced lower levels of cyclopropanated product (data not shown).

### Location of Cyclopropanated Fatty Acids in *L. infantum*

**Figure 5:**
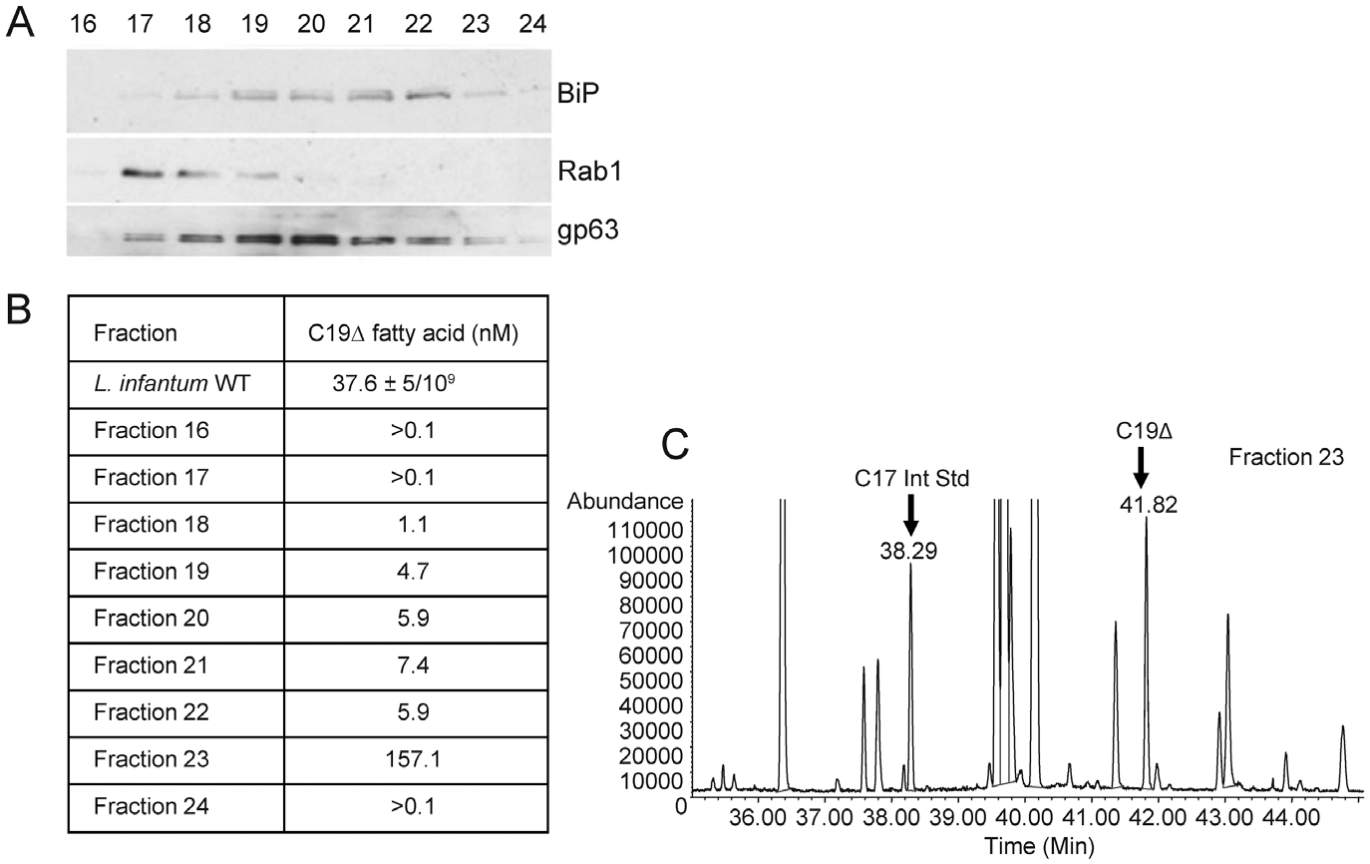
Sub-cellular localisation of cyclopropanated fatty acids in *L. infantum*. (A) Immunoblot analysis of sucrose gradient-separated sub-cellular fractions of wild type *L. infantum* promastigotes. Fractions 16–24 are shown, probed with antibodies specific for BiP, Rab1 and gp63. (B) GC-MS was used to determine the cyclopropanated fatty acid content of the fractions analysed in A, in comparison with wild type parasites. (C) Total ion chromatogram of derivatised fatty acid extracts from fraction 23. The spectral peak corresponding to the C19 cyclopropanated fatty acid (C19Δ) has a retention time of ∼42 min in this analysis.

To determine the localisation of the cyclopropanated fatty acid end products of CFAS activity, rather than the localisation of the enzyme itself, mid log phase parasite lysates were gradient-separated by ultracentrifugation and fractions analysed by immunoblotting and GC-MS. As shown in Figure 5, a spectral peak corresponding to CFAS-modified fatty acid (C19Δ) with a retention time of ∼41.8 min, was detected in Fractions 18–23 of the gradient, with >80% of the total localising in Fraction 23. (Note that the lower retention time, compared to Figure 4, was due to removal of a small section of the front of the column between runs; this led to a concomitant proportional drop in retention time of the internal standard C17 fatty acid). The separation of plasma membrane, ER and ER/cis-Golgi components through the gradient was detected using antibodies to GP63, BiP and Rab1 respectively. These data suggest that C19Δ fatty acids can be localized in both ER and plasma membrane fractions but most are found elsewhere in the parasite.

### In vitro Analysis of CFAS Null Parasites

**Figure 6:**
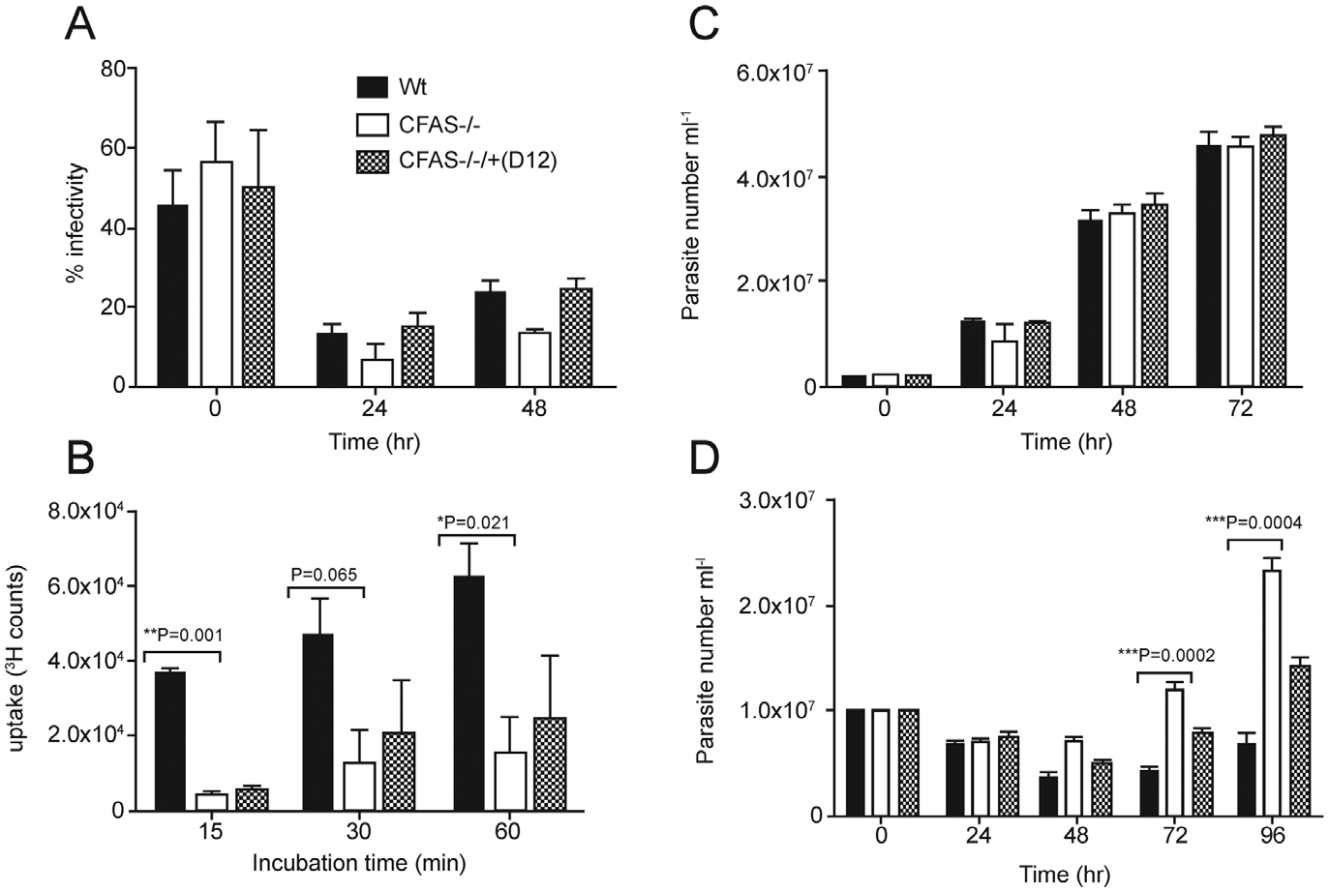
Phenotypic analysis of *L. infantum* CFAS mutants *in vitro*. (A) Bone marrow-derived macrophages were infected with late stationery phase wild type, CFAS null and complemented **CLN-D12** *L. infantum*, at a macrophage to parasite ratio of 1∶10. Numbers of infected and un-infected macrophages were counted (at least 200 macrophages per cell line at each time point) and the percentage infectivity calculated. (B) Proline uptake assay. The wild type, null and complemented *L. infantum* lines used in (A) were incubated with ^3^H-labelled L-proline and the internalized radiolabel quantified by liquid scintillation counting. Assays were performed in triplicate for each cell line. The wild type, null and complemented *L. infantum* used in (A) were cultured in M199 medium/20% FCS (C) or in the same medium supplemented with 300 µM hydrogen peroxide (D). Parasite growth rate over 72 hr (C) or 96 hr (D) was monitored by counting parasite numbers at each time point. Statistical differences was determined using the unpaired Student’s t-test with a value of P<0.05 considered significant. The same histogram shading, as shown in (A), is used in all panels of this figure to designate the different parasite lines.

To address the physiological role of CFAS fatty acid modification in *L. infantum*, a range of cell-based analyses were used to investigate the phenotype of parasites deleted for CFAS activity. Firstly, uptake and maintenance of wild type, CFAS null and CFAS complemented promastigotes in bone marrow-derived macrophages, as compared to wild type, were monitored over a time course (Figure 6A). At time point zero (after a 3 hr pre-incubation), all parasites lines showed similar levels of invasion (43–58% infectivity, with similar parasite numbers in each, data not shown) while these figures had fallen to less than 10% within 24 hr, indicative of macrophage killing, with no statistical difference between the different parasite lines. Numbers of infected macrophages at 48 hr, by which time differentiation to amastigotes had occurred, were also similar. These results suggest that CFAS null parasites are not significantly compromised in their early survival after phagocytosis.

Parasite survival both extracellularly in the vector and intracellularly in the host requires optimal plasma membrane integrity and function. To investigate generic loss of membrane function, we first chose to monitor membrane transporter activity, focusing on transport of the amino acid proline (found abundantly in the vector gut; [27]). CFAS null and CFAS complemented promastigotes, plus wild type parasites at the same growth stage, were incubated in the presence of radio-labelled L- proline and uptake assayed over a 60 min time course, using established methods (Figure 6B). Proline uptake was significantly higher in wild type parasites as compared to the CFAS−/− cell line, while the CFAS complemented parasites (**CLN-D12**, the same line used in all *in vitro* and *in vivo* experiments shown in this paper; Table 1) showed some increase in uptake activity, although not significantly more than the null mutant. These results are consistent with compromised transporter function in the absence of CFAS activity but the lack of robust complementation requires caution in this interpretation.

As another approach to analysing membrane function and molecular uptake in the CFAS mutants, we focused on endocytosis via the flagellar pocket, the main route by which many macromolecules enter kinetoplastid cells. CFAS null and complemented promastigotes, together with wild type parasites, were incubated in the presence of FITC- labelled concanavalin A (ConA-FITC) for 30 min or 2 hr at 26°C, prior to fixation and observation of the sub-cellular location of the fluorescent signal using confocal microscopy. Signal location was scored as exclusively at the flagella pocket (FP) or in the early endosomes (EE) after the 30 min or 2 hr incubation period respectively. No significant differences in the distribution of signal within the endocytic pathway were detected when comparing wild type and CFAS mutant cell lines over time (Figure S3). Taken together with the proline uptake data in Figure 6B, these analyses suggest that membrane modification due to the presence of cyclopropanated fatty acids has no effect on the endocytic pathway in *L. infantum* but may play a more generic survival role in a nutrient-poor environment by influencing transporter-mediated uptake.

We then tested whether the CFAS parasite lines could tolerate growth as promastigotes in the presence of a sub-lethal dose of hydrogen peroxide (300 µM). When compared to growth of the same lines in the absence of the oxidising agent (Figure 6C), growth of all the parasites lines was compromised at 24 hr of the 96 hr time course (Figure 6D). However, from 48 hr onwards, the CFAS null parasites were significantly more tolerant to hydrogen peroxide than the wild type or CFAS complemented parasites (Figure 6D). Interestingly, a similar phenotype has been observed in *L. mexicana* parasites null for cysteine proteinase C which, when exposed to 1 mM hydrogen peroxide for 60 min (a dose that induces cell death in these cells [28]), show better survival than wild type cells. The explanation of these data is currently unclear.

### *In vivo* Analysis of CFAS Null Parasites

**Figure 7:**
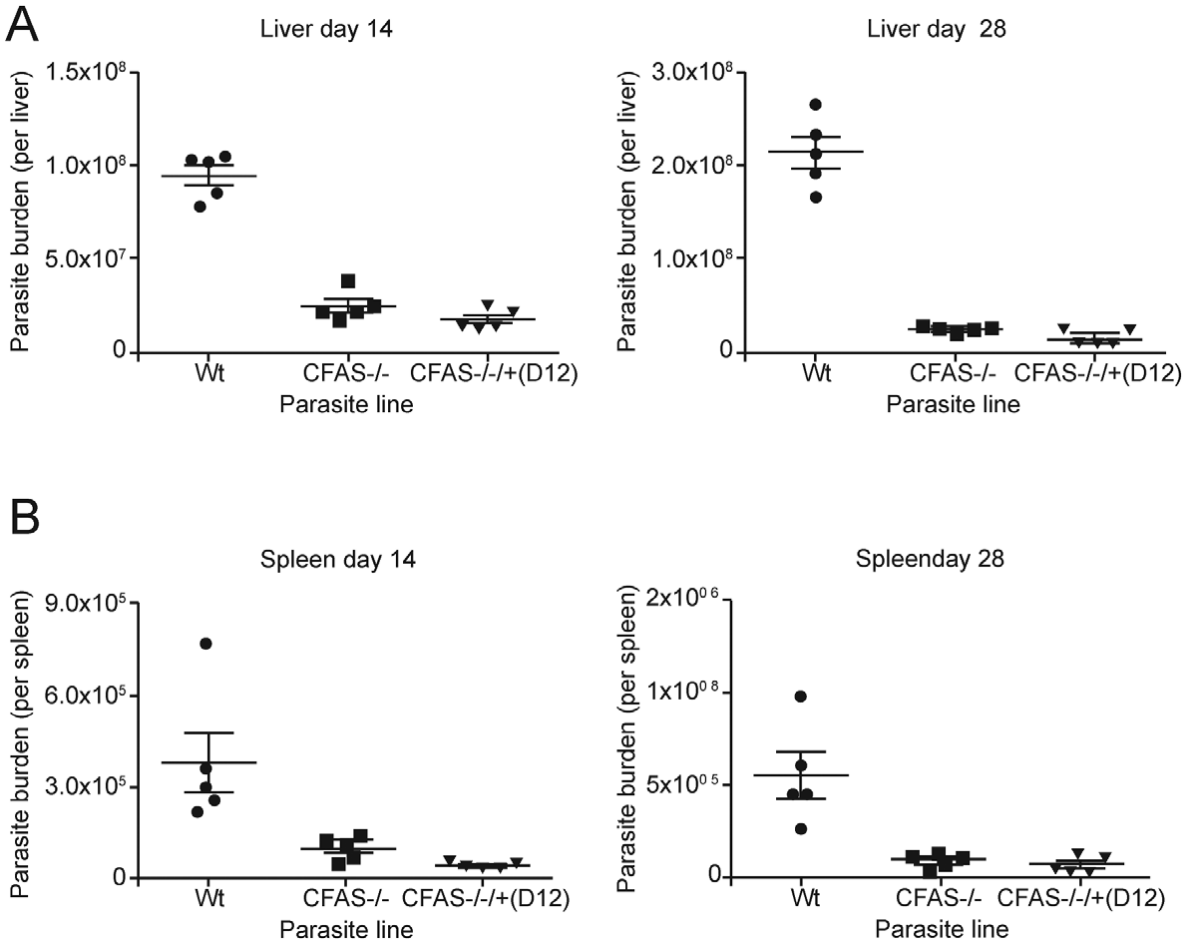
Survival of *L. infantum* CFAS mutants following infection *in vivo*. Groups of 5 BALB/c mice were infected intravenously with 2×10^7^ *L. infantum* wild type (Wt), CFAS null (CFAS−/−) and CFAS complemented **CLN-D12** parasites. Parasite numbers were determined in the liver (A) and the spleen (B) at 14 and 28 days post-infection (as described in Materials and Methods).

The role of CFAS in *Leishmania* pathogenesis *in vivo* was investigated using the same panel of wild type and CFAS mutant parasites as those used in Figure 6 and described in Table 1. Following intravenous (i.v.) infection, *L. infantum* CFAS −/− mutants were capable of establishing infection in the liver and spleen of BALB/c mice, indicating that this gene is not essential for initial parasite establishment in the mammalian host, in agreement with the macrophage infection data (Figure 6). However, the absence of CFAS in these mutants severely impaired *in vivo* survival, with a significant reduction in parasite burden observed in these organs (Figure 7). In the liver, the number of wild type *L. infantum* amastigotes increased 2.1 fold between days 14 and 28 post-infection (p.i.). By contrast, in addition to an initially lower parasite burden at day 14 p.i. (p<0.00001), parasite loads in CFAS null infected mice did not increase significantly between day 14 and day 28 p.i. (0.9 fold increase; p = ns; Figure 7A). In the spleen, parasite burden was lower than in the liver, in keeping with the organ-specific control of *L. infantum* [29]. Nevertheless, mice infected with CFAS null parasites showed significant reductions in parasite burden at day 14 p.i. (p<0.05) compared to mice infected with wild type parasites (Figure 7B). Thus the absence of CFAS resulted in significantly lower parasite burdens in both liver and spleen, perhaps indicative of impaired parasite capacity for replication or enhanced susceptibility to host killing.

To test complementation of this infectivity phenotype, three of the *L. infantum* CFAS-complemented cell lines were used for i.v. infection (**CLN-C2, CLN-D12, CLN-E7,** Table 1). We present here only the data generated with **CLN-D12** (the clone used in the experiments described in Figure 6) as all three clones gave very similar results. Although **CLN- D12** parasites produced <50% of the C19Δ fatty acid detected in wild type parasites (Table 1), parasite survival post-infection with this complemented line was severely affected, with significantly reduced burdens in both the liver and spleen (Figure 7). Similar results were obtained using **CLN-E7**, which produced ∼75% of the wild type level of C19Δ fatty acid, while **CLN- C2**, producing ∼350% of the wild type C19Δ fatty acid level, also showed significantly reduced parasite burden *in vivo* (data not shown). In summary, all clones tested by this analysis were compromised in their infectivity and none complemented the wild type *L. infantum* phenotype *in vivo*, although all grew as wild type promastigotes in culture (as in Figure 3). Thus, whether the *in vivo* infectivity defect observed in the CFAS nulls is the result of loss of CFAS activity remains inconclusive due to this lack of robust complementation.

**Figure 8:**
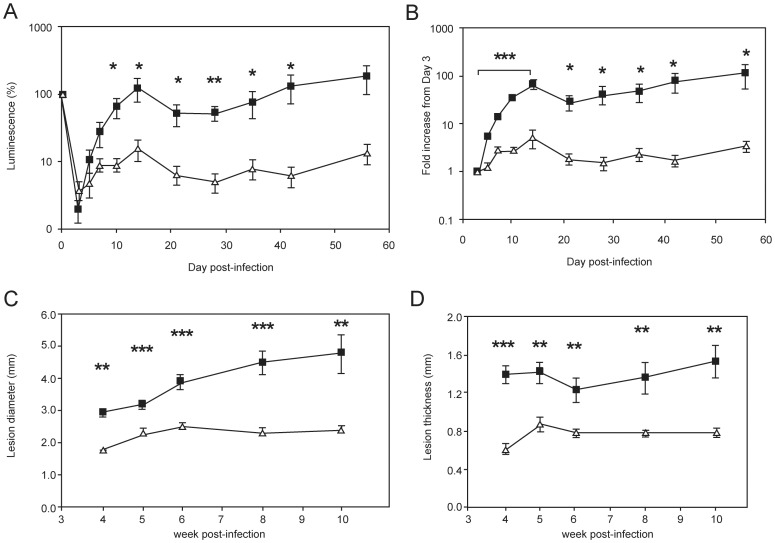
CFAS expression attenuates *L. major* dermal infection *in vivo*. BALB/c mice were infected intradermally with 1×10^6^ *L. major* LUC (closed squares) or *L. major* LUC+CFAS (**CLN-4**, open triangles) and parasites were visualised by bioluminescence imaging using an IVIS over the course of the infection. Luciferase activity (photons/second) is expressed as (A) a percentage change over time and (B) as a fold increase from day 3 post-infection. Lesion progression was monitored by measurement of lesion diameter (C) and thickness (D) from week 4 onward. * p<0.05, ** p<0.01, *** p<0.001, by unpaired Student’s t-test (n = 5).

**Figure 9:**
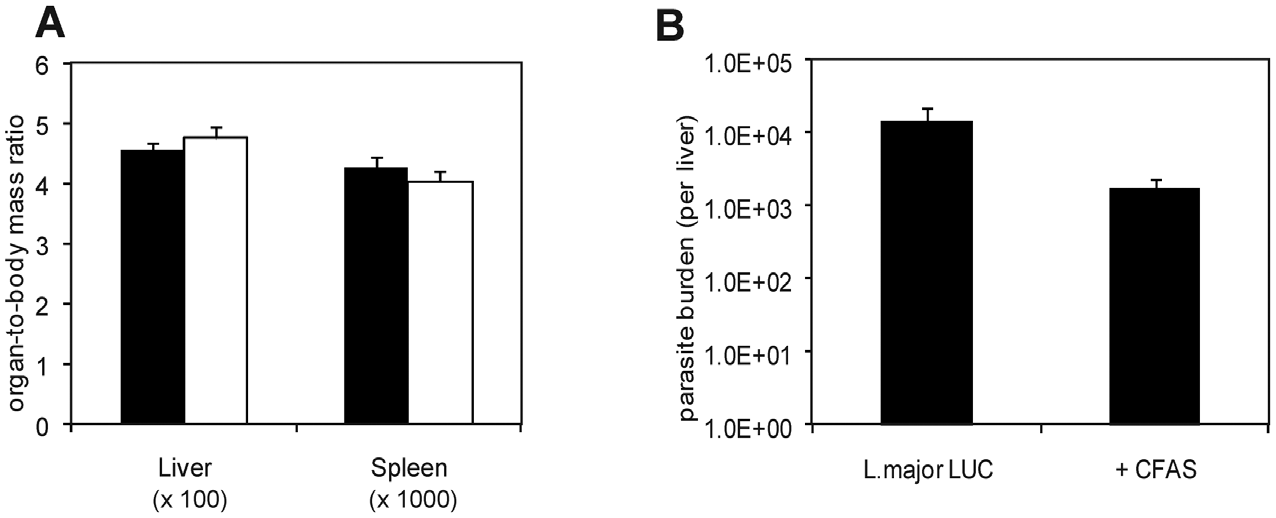
The expression of CFAS does not enhance viscerotropism of *L. major*. BALB/c mice were infected with *L. major* LUC (black bars) or *L. major* LUC+CFAS (white bars) and (A) hepatomegaly and splenomegaly and (B) parasite burdens were determined at Day 70 post-infection. Parasite clones were those used in Figure 8. p = 0.07 by unpaired Student’s t-test (n = 5).

As an alternative approach to investigating the functional significance of cyclopropanation in *Leishmania* species, transgenic *L. major* parasites expressing CFAS (which they do not normally produce) were generated and characterised (Table 1, Figure S2). To facilitate non-invasive evaluation of parasite burden *in vivo*, we generated these lines using a parental *L. major* strain expressing luciferase (LUC). CFAS-expressing and wild type *L. major* LUC lines had equivalent luciferase activity both *in vitro* (data not shown) and after injection intradermally into BALB/c mice, as monitored using biophotonic imaging. Following *in vivo* infection, ectopic expression of CFAS resulted in a significant attenuation of parasite virulence (Figure 8, Figure S4). Relative to baseline infection levels (determined at 4h post injection), tissue luminescence had significantly decreased by day 3 p.i. (Figure 8A). This loss of bioluminescence signal may be due to death of parasites and/or differential luciferase activity during the differentiation from infective promastigotes to intracellular amastigotes. The latter interpretation is supported by the observation that *L. major* LUC amastigotes purified from chronic lesions had a more than 10-fold decrease in luciferase activity on a cell per cell basis compared to promastigotes (Figure S5). It has been suggested that this may indicate decreased transcription from the ribosomal locus during the amastigote life cycle stage [30]. To adjust for this variation, we also normalised our data by determining the fold increase at each time point relative to the bioluminescence signal at day 3 p.i. As shown in Figure 8B, *L*. *major* LUC parasites showed a sustained increase in luminescence over the first two weeks of infection, which resulted in a 67-fold increase in signal intensity measured at day 14 p.i. In contrast, bioluminescence signal increased around 5-fold after 14 days of infection with CFAS-expressing *L. major* LUC and returned to baseline (day 3) levels by day 21 p.i. (Figure 8B). Attenuation of virulence due to CFAS expression was also directly observed by scoring lesion severity in terms of both lesion diameter (Figure 8C) and thickness (Figure 8D). Of note, expression of CFAS in the *L. major* parent line did not influence parasite tissue tropism. Parasite burden in the liver was minimal, parasites were undetectable in the spleen (data not shown) and there was no evidence of hepato-splenomegaly in CFAS-expressing *L.major* parasites (Figure 9).

## Discussion

Cyclopropanated fatty acids have been identified in a range of organisms, including bacteria, parasitic protozoa, fungi and plants [13], [31], [32]. However, the cyclopropane fatty acid synthetases which catalyse the generation of cyclopropane rings have only been extensively studied in the two bacterial species, *E. coli* and *M. tuberculosis*. In *E. coli*, the CFAS reaction mechanism has been studied in detail, using chemical modification and site-directed mutagenesis to identify key residues for catalysis [26]. It has been estimated that formation of one cyclopropanated ring requires 3 molecules of ATP, a high energy requirement suggesting that this modification confers some unique advantage for survival to cells that carry cyclopropanated lipids [13]. Indeed, studies in *E. coli* have shown that increases in CFAS activity, leading to increased cyclopropanated fatty acid content, are associated with changes in environmental conditions such as exposure to high temperature, low pH, high salt concentration and depressed oxygen tension [33], [34], further supporting the proposal that this modification functions as a cellular survival mechanism. For example, *Helicobactor pylori,* which colonizes the mammalian gut and is associated with reduced gastric acidity, secretes large amounts of cyclopropanated fatty acid (*cis* 9, 10-methyleneoctadecanoic acid) in contrast to other bacterial species that also colonize the intestinal tract [35]. The cyclopropanated fatty acids produced by the gastric colonizers have been shown to be active in inhibiting the gastric H^+^/K^+^-ATPase proton pump, leading to reduced acidity in the infected regions [36].

In *Mycobacteria*, cyclopropanation of mycolic acids is common among the pathogenic species but rare in non-pathogenic species. *M. tuberculosis* expresses a family of eight related enzymes sharing sequence identity with *E. coli* CFAS [11]. Six of these catalyze the transfer of a methyl group from SAM to the double bond of the unsaturated mycolic acyl chains [37]. Mycobacterial cyclopropanated fatty acid structure is more complex than that found in *E. coli*: the mycolic acids are cyclopropanated at two positions, distal and proximal with respect to the position of the functional head group of the acyl chain. Furthermore, the cyclopropane ring can be in the *cis* or *trans* position, with the enzymes showing stereo-specificity in their activity. This structural diversity, coupled with dynamic modifications that occur within the cell envelope, is known to play an important role in the resistance of *M. tuberculosis* to antibiotics, dehydration and low pH within the macrophage phagolysosome [38]. In addition, cyclopropane modification of trehalose dimycolates has been demonstrated to modulate host cell immune activation during *M. tuberculosis* infection [12], [39], with specific *cis*-cyclopropanation shown to be essential for establishment of chronic persistent infection in mice. Conversely, *trans*-cyclopropanation causes suppression of *M. tuberculosis*-induced inflammation and virulence [12], [39]. These observations have encouraged recent genetic and chemical biology analyses, suggesting that mycolic acid methyltransferases are potential targets for antibiotic development [40].

In the Kinetoplastida, early biochemical analysis detected cyclopropanated fatty acids in some but not all species. This work showed that most species of the genera *Crithidia*, *Leptomonas*, *Herpetomonas* and *Phytomonas* contain these modified fatty acids but failed to detect them in *Trypanosoma*, *Blastocrithidia* or *Endotrypanum* species [41]. This pattern of distribution did not provide any immediate clues to inform functional analysis at the time. More recently, genomic sequencing in the genus *Leishmania* has identified a single CFAS gene in *L. infantum*, *L. braziliensis* and *L. mexicana* [5], [7] that is missing in *L. major,* suggesting that CFAS function is no longer required in this species.

Here, we present the first functional characterisation of CFAS in pathogenic *Leishmania* species, focusing on *L. infantum*, causative agent of VL. We have cloned *L. infantum CFAS* and confirmed its presence as a single copy gene that is constitutively expressed in both extracellular and intracellular parasite stages, with higher levels of stable mRNA detected in intracellular amastigotes (Figure 1). In extracellular promastigotes, the CFAS protein, when expressed from a tagged transgene, is only stable in early to mid logarithmic phase and is rapidly degraded as cells enter late logarithmic and stationary phases of growth in culture. Conversely, tagged CFAS protein can be detected in amastigotes following macrophage infection (Figure 2). Using mutant parasites, we show that *L. infantum* CFAS is functional in the cyclopropanation of fatty acids in *L. infantum*, activity that is lost in parasites null for the CFAS gene but restored following genetic complementation (Figure 4). Heterologous expression analysis also shows that *L. infantum* CFAS can catalyse cyclopropanation in *L. major* parasites, generating the characteristic cyclopropanated C19Δ fatty acid product. This observation suggests that the C18:1 fatty acid substrate may be ubiquitous in *Leishmania* species, while its modification by cyclopropanation is a species-specific property. The accumulation of cyclopropanated product in an as-yet-unidentified subcellular compartment in *L. infantum* (Figure 5) may aid identification of the CFAS substrate in promastigotes. One possible location is in association with a mitochondrial-associated ER membrane complex (or MAM complex) which is critical for lipid transport in mammalian cells but has not yet been characterised in kinetoplastids [42]. Given the localisation of the CFAS enzyme to the ER (Figure 2A), the fraction of cyclopropanated fatty acids detected in this location may represent newly synthesised product prior to trafficking to the plasma membrane, possibly via a MAM-like complex.

The cellular location of the CFAS enzyme in bacteria has not been conclusively demonstrated, although earlier studies in *E. coli* suggested a cytosolic location with some peripheral attachment to the inner membrane [43]. The question of where and how the enzyme gains access to its fatty acid substrate has not been resolved, however, and it is unknown whether the fatty acid or lipids are modified *in situ* or during synthesis, prior to incorporation into membranes. The ER localisation of *L. infantum* CFAS correlates with recent reports on fatty acid synthesis in the related kinetoplastid species, *Trypanosoma brucei*, which describe an unconventional mechanism of *de novo* fatty acid synthesis that involves the use of ER-located microsomal elongases [44], [45]. Studies of CFAS in the plant species, *Sterculia foetida,* also localize CFAS to microsomal membranes [31]. Although most trypanosomatids are devoid of CFAS, the subcellular location of the *Leishmania* enzyme in the ER suggests that the fatty acid substrates are probably cyclopropanated during or immediately after their synthesis at this subcellular location.

Deletion of the *L. infantum* CFAS gene or its over-expression in “add-back” lines did not affect parasite growth *in vitro* (Figure 3). Similarly, heterologous over-expression of *L. infantum* CFAS in *L. major* had only a minor impact on growth *in vitro*. These observations suggest that fatty acid cyclopropanation is not essential for propagation of promastigotes, although this modification could be required for parasite transmission in sand flies. Of relevance here, the observed decreased uptake of proline in the CFAS nulls might influence viability in the proline-rich gut of the vector (Figure 6). The other phenotypic features of CFAS null promastigotes (resistance to oxidative stress but phagocytosis equivalent to wild type parasites; Figure 6) would support establishment of these cells in the macrophage phagolysosome. More generically, while modification by cyclopropanation has been proposed to alter membrane fluidity, our attempts to assay the biophysical properties of promastigote membranes using anisotropy showed no statistically significant differences between wild type, CFAS null and complemented parasites.

In contrast to most of the work *in vitro*, a distinct phenotype associated with loss of cyclopropanation in *L. infantum* amastigotes was observed using a range of mutant clones in a murine infection model (Figure 7). *In vivo* infectivity studies in susceptible BALB/c mice indicated that CFAS loss severely affected *L. infantum* virulence, as judged by liver and spleen parasite loads, reflecting compromised parasite replication and survival in both organs. However, complementation of this phenotype by restoration of wild type levels of enzyme activity could not be achieved. While the lack of a tightly-regulatable promoter system to control gene expression in *Leishmania* has held back generation of a complemented clone re-expressing CFAS to wild type levels, the “add-back” lines tested produced <50%, 75% and up to 350% of wild type cyclopropanated product (CLN- D12, CLN- E7 and CLN-C2 respectively, Table 1) with no restoration of phenotype. One interpretation of these data is that irreversible changes in cell physiology occur in CFAS null amastigotes that cannot be rescued by re-expression, a hypothesis that requires further investigation.

As an alternative approach, we expressed CFAS in *L. major* parasites, which normally lack cyclopropanated fatty acids, and this significantly compromised their virulence *in vivo,* as indicated by decreased lesion severity following dermal infection. The levels of C19Δ produced by the CFAS-expressing *L. major* parasites ranged from ∼6–12 fold over-expression as compared to *L. infantum* wild type levels, however, depending on the site of insertion of the transgene into the parasite ribosomal locus and its subsequent expression from the ribosomal promoter. It is therefore possible that this reduced severity of lesion formation could be due to C19Δ over-production, a phenotypic effect similar to that observed in the *L. infantum* over-expressing mutants. More significantly, there was no alteration in parasite burdens in liver and spleen after long term infection with *L. major* expressing CFAS, demonstrating that whatever the short-term influence of aberrant cyclopropanation in this parasite species, the presence of modified fatty acids does not influence parasite tropism in long-term infection.

While the physiological role of cyclopropane modification has not been fully defined in any species, the expression of CFAS in many bacteria and the sporadic distribution of the gene in a few phylogenetically unrelated eukaryotes [31], [32], [41] suggest that different organisms use this modification to facilitate adaptation to environmental conditions or undergo key developmental processes requiring changes in membrane structure and function. Cyclopropanation of fatty acids has been associated with drought tolerance in plants [46], egg development in millipedes [47] and fruiting body initiation in the fungus *Coprinopsis cinerea* [32], while in bacteria, the modification has consistently been linked to acid tolerance [13]. For intracellular pathogens, cyclopropanation may play a role in survival in physiologically hostile and nutrient-poor compartments within the host cell. This would be of particular relevance to *Leishmania* species which, unlike those pathogens that avoid mature phagolysosomes in macrophages, have the capacity to survive and replicate in these acidic and nutrient-poor compartments. For survival, the parasite has to tolerate macrophage antimicrobial effectors such as reactive nitrogen and oxygen species, or find ways of subverting normal cellular mechanisms in order to avoid killing. One mechanism could be via generation of cyclopropanated fatty acids which can inhibit H+/K+-ATPases [36] and facilitate modifications required for survival in acidic environments [33], [48]. Why only *L. major* of the *Leishmania* species currently analysed has lost the CFAS gene and does not produce this enzyme remains unknown but may suggest that biological aspects of the intracellular survival of this species are uniquely specialised.

## Materials and Methods

### Leishmania Culture and Generation of Mutant Parasites

The two sequenced genome reference strains used were *L. major* MHOM/IL/80/Friedlin and *L. infantum* clone JPCM5 MCAN/ES/98/LLM-877 [5], [6]. Promastigotes were cultured at 26°C in modified Eagle’s medium (HOMEM; *L. infantum*) or 1 x M199 medium (*L. major*) supplemented with 10% heat-inactivated foetal calf serum (FCS, Invitrogen) and penicillin-streptomycin (Invitrogen). Amastigotes were isolated from infected tissues as described [51]. Transfected parasites were maintained in the following antibiotics as required: hygromycin at 32 µg/ml, puromycin (Calbiochem, La Jolla, CA) at 20 µg/ml, and neomycin (G418, Geneticin; Life Technologies) at 20 µg/ml or 40 µg/ml when grown in liquid medium. Growth phenotypes were analysed by culturing cell lines in the absence of drug selection and counting cell densities in triplicate at time intervals using a Coulter Counter (Beckman Coulter).

*L. infantum* CFAS null mutants were generated by sequential transfection with hygromycin (HYG) and puromycin (PUR) knockout (KO) constructs, based on the pX63-HYG plasmid vector [49], using DNA flanking sequences immediately upstream (558 bp) and downstream (263 bp) of the CFAS open reading frame (ORF) as targets for homologous recombination. These fragments were amplified by PCR and cloned to flank the HYG gene generating the first allele KO construct, pX63-HYG-CFAS-KO. For deletion of the second allele, HYG was replaced by PUR, generating the targeting construct pX63-PUR-CFAS-KO. The following oligos were used for amplification and cloning (with restriction sites underlined): CFAS-upstream-F, 5′-*GC* aagcttatacgtacgcagaggcatcgg-3′, CFAS-upstream-R, 5′-*AT* gtcgaccatgcttggccggagcaacg-3′, CFAS-downstream-F, 5′-*GT* cccgggttgcatcggcgtggctgagc-3′, CFAS-downstream-R, 5′-*CT* agatctagacgccgacgcaggcattc-3′.

Constructs were digested with *Hin*dIII and *Bgl*II to release the linear KO cassettes which were used for transfection of mid-log phase *L. infantum* promastigotes as previously described [49].

To complement CFAS KO or ectopically express CFAS in *L. infantum* and *L. major* parasites, constructs were made based on the pSSU-int vector [50] carrying neomycin (NEO) resistance instead of HYG. The CFAS ORF was amplified by PCR and cloned, using *Xho*I/*Bam*HI sites, into the modified pSSU-int vector, generating pSSU-Neo-CFAS. For expression of C-terminal myc or HA-tagged CFAS proteins, the reverse complement of the myc (5′-GGATCC*ttacaggtcttcttcagagatcagtttctgttc*-3′) or HA (5′-GGATCC*ttaggcatagtccgggacgtcgtaggggta*-3′*)* tag sequence (italics) including the stop codon, were fused to a 18 nt CFAS reverse primer (5′-cggccggtacacgctgac-3′) without the stop codon. The resulting primer was used together with a CFAS forward primer (5′-CTCGAGatggaaaaccggccacacga-3′) to amplify the CFAS ORF, for subsequent cloning into the *Xho*I/*Bam*HI sites of pSSU-int, generating pSSU-CFAS-myc or pSSU-CFAS-HA. For *L. major* transfection, pSSU-CFAS was digested with *Pac*I and *Pme*I, releasing a linear fragment for targeting into the ribosomal locus. For *L. infantum* transfections, the 5′SSU sequence was replaced (following *Nde*I/*Xho*I digestion) with its orthologue amplified from the *L. infantum* genome and the resulting construct (pSSU-Inf-CFAS) linearised with *Nd*eI and *Pme*I prior to transfection. Complementary CFAS KO cell lines were also generated using constructs that facilitate expression of an add-back CFAS gene from the endogenous locus. These constructs were generated by replacing the 5′ and 3′ SSU integration sequences in pSSU-Inf-CFAS-HA vector with CFAS 5′ and 3′ untranslated region (UTR) sequences respectively.

### Isolation and Analysis of Nucleic Acids

Total *Leishmania* RNA was isolated using TRIZOL (Invitrogen, according to the manufacturer’s instructions) and dissolved in RNAse-free water. *Leishmania* genomic DNA was isolated using a DNAeasy kit (Qiagen) as described [52]. Southern blot techniques were used to analyse CFAS KO cell lines or integration of an ectopic copy into the ribosomal locus for expression in either *L. infantum* or *L. major*. For CFAS KO analysis, genomic DNA was digested with *Sal* I and fragments size-separated by electrophoresis using 0.8% agarose. For CFAS ectopic expression, integration into the ribosomal locus was analysed by *BamH* I digestion of genomic DNA, followed by fragment separation through 1% agarose in 0.5 x TBE, using a CHEF pulsed-field gel electrophoresis (PFGE) system (Bio-Rad) set at 150 V, with initial and final switching times of 10 sec each and a run time of 20 h at 25°C. Fragments were blotted and hybridised with a 464 bp (nt 532–996) CFAS specific DNA probe prepared using a Digoxigenin-labelling and PCR DIG probe synthesis kit (Roche). Blots were prehybridised for 4 hr in DIG Easy Hyb buffer (Roche), hybridized at 42°C overnight in the same buffer containing 20 ng/ml of DIG-labelled probe, washed twice in 2 × SSC, 0.1% SDS at room temp for 5 min and then twice in 0.5 × SSC, 0.1% SDS at 68°C for 15 min. Hybridisation was detected using anti-DIG antibody conjugated to alkaline phosphatase and CDP-Star substrate (Roche).

### Expression Profile and Parasite Burden Analyses Using Quantitative Real-Time PCR

For expression analysis, total parasite RNA was DNase-treated (Ambion, according to the manufacturer’s instructions) prior to reverse transcription into cDNA using an Omniscript RT kit (Qiagen), Oligo dT (Promega), and RNase inhibitor (Promega) according to the manufacturers’ instructions. RT-qPCR was performed with an ABI Prism 7000 Sequence detection system (Applied Biosystems) using SybrGreen and primers designed according to Primer Express software and guidelines (Applied Biosystems). Expression levels of target genes were quantified relative to housekeeping gene elongation factor I-α (EFI-α; LinJ17_V3.0090).

Parasite burdens in liver and spleen tissues, sampled in duplicate, were assessed using RT-qPCR (as described in [51]). DNA standards were created for each tissue sampled, by spiking naïve tissue with known numbers of *L. infantum* promastigotes and analysing the extracted DNA as described. Total parasite burden per organ was calculated by determining the total number of parasites per weight (in grams) of tissue sampled and multiplying by total organ mass.

### Fluorescence Microscopy

Parasites were fixed in 4% paraformaldehyde for 15 min at room temp, washed three times with PBS before depositing on to polylysine–coated slides. Parasites were permeabilised for 10 min in PBS containing 5% FCS and 0.1% Triton X-100, blocked for 15 min in PBS containing 5% FCS before staining with monoclonal rat anti-HA antibody (F10, Roche Diagnostics) or rabbit anti-BiP antibody (the gift of Jay Bangs). Alexa Fluor 546- and 488- conjugated goat anti-rat or rabbit IgG were used as secondary antibodies. Stained cells were mounted with Vector Shield containing DAPI before viewing on a fluorescent or confocal Zeiss LSM 510 meta microscope with a Plan-Apochromat 63x/1.4 Oil differential interference contrast I objective lens. Images were acquired using LSM 510 version 3.2 software (Carl Zeiss, Jena, Germany) as described previously [53].

### Subcellular Fractionation and Immunoblotting

Parasites were washed twice in PBS pH 7.4, resuspended at 1×10^8^ cells ml^−1^ in hypotonic buffer (1 mM potassium acetate, 1.5 mM magnesium acetate, 1 mM CaCl_2_, 10 mM Tris, 2 mM EDTA, pH 7.2) containing protease inhibitors (complete mini EDTA-free protease inhibitor cocktail, Roche Diagnostics) and lysed by sonication on ice for 4×15 sec bursts (40 watts). The lysate was centrifuged at 1000 rpm for 2 min at 4°C to remove non-lysed cells. The resulting supernatant was centrifuged at 83,689 g (45000 rpm, Beckman TLA-100.3 rotor) for 90 min at 4°C to separate membrane and cytosolic components [22]. For sucrose gradient fractionation, cells were processed as described [54]. Briefly, 1×10^9^ *L. infantum* promastigotes suspended in hypotonic buffer (2 mM EGTA, 2 mM DTT, 2 mM leupeptin, 0.1 mM phenylmethylsulfonyl fluoride [PMSF]), were lysed by expulsion (×15) through a 27 gauge needle. The lysate was made isotonic by addition of 4× assay buffer (50 mM HEPES-NaOH pH 7.4, 0.25 M sucrose, 1 mM ATP, 1 mM EGTA, 2 mM DTT, 2 mM leupeptin, 0.1 mM PMSF) and centrifuged at 3000 g for 10 min. The supernatant was transferred to a clean tube and kept at 4°C prior to gradient separation. Sucrose gradients were prepared by layering 10×0.8 ml fractions of 0.25–2 M sucrose (in 25 mM HEPES–NaOH, pH 7.4) over a 2.5 M sucrose cushion in Ultraclear Centrifuge tubes (Beckman) and centrifugation at 218 000 g for 1 hr. Cell lysate supernatant was then layered on top of the linear sucrose gradient and fractionated by centrifugation at 218 000 g for 6 hr at 4°C (Beckman L-60 Ultracentrifuge, SW41Ti rotor). Fractions (0.5 ml) were collected from the bottom of the tube and analysed by immunoblotting as described [55], probing with either rat anti-HA (Roche), mouse anti-myc (Invitrogen), rabbit anti-BiP, rabbit anti-HASPB [18], rabbit anti-Rab1 or mouse anti-GP63 (the gift of Robert McMaster) followed by ECL detection (Amersham Biosciences).

### Identification and Quantification of Cyclopropane-containing Fatty Acids

Parasite fatty acids were characterised and quantified by derivatisation to their fatty acid methyl esters (FAME) followed by gas chromatography-mass spectrometry analysis. Briefly, mid-log phase *Leishmania* were collected by centrifugation, washed in PBS and freeze-dried in glass tubes. Triplicate aliquots (equivalent to 2×10^8^ cells) were transferred to 2 ml glass vessels, spiked with an internal standard fatty acid C17:0 (20 µl of 1 mM) and dried under nitrogen. Base hydrolysis to release fatty acids was performed using 500 µl of concentrated ammonia and 50% propan-1-ol (1∶1), followed by incubation for 5 hr at 50°C. After cooling, samples were evaporated to dryness with nitrogen and dried twice more from 200 µl of methanol: water (1∶1) to remove all traces of ammonia. The protonated fatty acids were extracted by partitioning between 500 µl of 20 mM HCl and 500 µl of ether. The aqueous phase was re-extracted with fresh ether (500 µl) and the combined ether phases were dried under nitrogen in a glass tube. The fatty acids were converted to FAME, by adding diazomethane (3×20 µl aliquots) to the dried residue, while on ice. After 30 min, samples were allowed to warm to room temp and left to evaporate to dryness in a fume hood. The FAME products were dissolved in 10–20 µl dichloromethane and 1–2 µl analysed by GC-MS on Agilent Technologies (GC-6890N, MS detector-5973) with a ZB-5 column (30 M × 25 mm × 25 mm, Phenomenex), with a temp program of 70°C for 10 min followed by a gradient to 220°C at 5°C/min and held at 220°C for a further 15 min. Mass spectra were acquired from 50–500 amu. The identity of FAMEs was carried out by comparison of the retention time and fragmentation pattern with a bacterial FAME standard that contained both C17Δ and C19Δ (Supelco).

### In vitro Cellular Assays

Proline uptake was assayed following published procedures [56], [57], with minor adaptations. Mid log phase promastigotes were washed twice in PBS, pH 7.4 at 4°C and resuspended at a density of 1×10^8^ cells. ml^−1^. After 10 min pre-incubation at 30°C, 20 µl of L-[2,3-^3^H]-proline in PBS (0.2 mCi/ml, Perkin-Elmer) was added to 400 µl of cell suspension and uptake measured over a 60 min time course at 30°C. Uptake was terminated by centrifuging the parasites at 800 g for 5 min and washing the pellet 3 x in cold PBS. Incorporated radioactivity in the cell lysates was measured by liquid scintillation counting (TopCount.NXT™ Microplate Scintillation and Luminescence Counter; Packard Bioscience).

Bone marrow-derived macrophages (BMD) were isolated from BALB/c mice bred in pathogen-free conditions at the Centre for Immunology and Infection, University of York. All animal work was conducted under UK Home Office Licence requirements and after institutional ethical review. BMD were cultured at 37°C in Complete DMEM (Dulbecco's modified Eagle's medium supplemented with 10% foetal bovine serum (FBS), 2 mM L-glutamine, 100 units/ml penicillin G and 100 ug/ml streptomycin). Macrophages were plated at a density of 5×10^4^ cells per well in a 24-well plate containing glass cover-slips, allowed to adhere for 3 hr, then washed 3 times with DMEM to remove any non-adherent cells. Late stationery phase *L. infantum* (at 2.5×10^6^ cell/ml; each parasite cell line analysed in triplicate) were added to each well and the plate centrifuged at 1500 rpm for 10 min before incubation at 37°C for 3 hr. After incubation (time point zero), wells were washed twice to remove unattached parasites, fresh Complete DMEM (500 µl) added to each well and incubation continued at 37°C. Samples were collected at 0, 24 or 48 hr by washing the wells 3 times with cold PBS, fixing in 100% methanol for 5 min and staining with May-Grünwald-Giemsa, prior to microscopy to identify internalized parasites. Parasite infectivity was calculated by counting the number of infected macrophages as a % of total macrophages, counting at least 200 macrophages per cell line at each time point.

Tolerance to reactive oxygen species (ROS) was assessed by culturing log phase parasites in HOMEM complete medium supplemented with hydrogen peroxide (Sigma) at a final concentration of 300 µM. Parasite numbers were determined at 24 hr intervals, as described above.

Endocytosis assays were performed as described [58], [59]. Briefly, 1×10^7^ cells were harvested and washed once with 1 ml of serum-free medium. Cells were resuspended in 500 µl of serum-free medium containing 1% fat-free BSA (Sigma) and incubated for 30 min at 26°C. Fluorescein isothiocyanate-labelled lectin concavalin A (FITC-ConA; Invitrogen) was added to a final concentration of 5 µg/ml and incubation continued for 30 min or 2 hr. After incubation, cells were washed twice in PBS and fixed with 4% paraformaldehyde at 4°C for 1 hr. The fix was washed off twice with PBS before mounting for fluorescent microscopy analysis as described above.

### In vivo Infections

BALB/c mice (Charles River UK Ltd., Margate, UK) were housed in pathogen-free conditions in individual micro-isolators at the University of York. All animal work was conducted under UK Home Office License, after institutional ethical review. To reduce any impact of long term *in vitro* culture on parasite virulence, all parasite lines used for infections were passaged *in vivo* and subject to amastigote to promastigote conversion in *in vitro* liquid culture. All lines were used at comparable *in vitro* passage number (always less than 5). *L. infantum*, *L. infantum* CFAS−/− and *L. infantum* CFAS−/−/+ CFAS metacyclic promastigotes were purified on a Percoll gradient from late stationary phase *in vitro* cultures [60]. Infection-competent metacyclic parasites were gauged by morphology and expression of metacyclic specific HASPB protein (data not shown). *L. infantum* infections were established by intravenous (i.v.) infection with 2×10^7^ parasites and mice were killed at 14 and 28 days post infection (n = 5 per group). Parasite burdens in liver and spleen tissues were determined as described above.

*L. major* infections were established by intradermal infection into the ear pinnae with 1×10^6^ metacyclic promastigotes purified on a Ficoll gradient from late stationary phase *in vitro* cultures [61]. *L. major* LUC or *L. major* LUC+CFAS inoculums showed equivalent levels of luciferase activity *in vitro* (data not shown). Baseline levels of luciferase activity were taken *in vivo* at 4 hr post infection. Cutaneous lesions were assessed at various time points using vernier callipers. Mice were sacrificed 10 weeks post infection and tissue parasite burdens determined by qPCR as described above.

### Bioluminescence Imaging with IVIS

Mice were anesthetised by isoflurane inhalation, and injected intraperitoneally (i.p.) with D-luciferin at 150 mg/kg. Bioluminescence images were acquired at 20 min post-injection with a 5 minute exposure using an IVIS Imaging 100 system (Xenogen Corp.). Analysis and acquisition were performed using Living Image software, version 2.5 (Xenogen). In brief, luminescence (photons/second) was determined in a manually defined region of interest (ROI) over the ear pinnae. Background readings from a comparable ROI over the contra-lateral ear pinnae were subtracted from each measurement. For determination of luciferase activity *in vitro*, *L major* LUC promastigotes were obtained from a low passage stationary phase *in vitro* culture, and *L major* LUC amastigotes were purified from a chronically infected BALB/c footpad. Luciferase activity was measured on a luminometer using a Luciferase Assay System kit (Promega) as per manufacturer’s instructions.

## Supporting Information

Figure S1 CFAS amino acid sequence analysis. AlignX (Vector NTI tool) was used to align *Leishmania* CFAS sequences with those from other species. A structurally conserved S-adenosyl-*L*-methionine (SAM) binding domain is underlined while other highly conserved residues are highlighted. Sequence accession number of the sequences used: XP_001463394 (*Leishmania infantum*); XP_001562118 (*Leishmania braziliensis*); NP_334895 (*Mycobacterium tuberculosis*-1); NP_215159 (*Mycobacterium tuberculosis*-2); AAC44617 (*Mycobacterium tuberculosis*-3); NP_215157 (*Mycobacterium tuberculosis*-4); NP_207214 (*Helicobacter pylori*); NP_416178 (*Escherichia coli*); AAL73238 (*Coprinopsis cinerea*); AAM33848 (*Sterculia foetida*); NP_188990 (*Arabidopsis thaliana*).
(TIF)

Figure S2 Analysis of CFAS transgene integrations into the ribosomal locus of *L. major.* (A) Map of the ribosomal integration vector construct (pSSU-NEO-CFAS) and the corresponding region of *L. major* chromosome 27 with repeated integration sites (SSU) distributed across the locus. The position of the ribosomal promoter (R) is indicated. (B) Southern blot analysis of genomic DNA extracted from wild type (wt) and transgenic *L. major* mutants expressing CFAS (**CLN-2, CLN-3**, Table 1). DNA was digested with *BamH* I, separated by pulsed field gel electrophoresis through 1% agarose, blotted and probed with the CFAS-specific probe shown in Figure 3A.
(TIF)

Figure S3 Endocytosis assay. The wild type, null and complemented *L. infantum* lines used in Figure 6A were incubated with FITC-labeled Con A and uptake stopped after 30 min or 2 hr by fixation with 4% paraformaldehyde. Analysing 100 parasites from each line, the number of parasites with Con A signal at the flagella pocket (FP) or in the early endosomal (EE) regions was counted and the percentage of the total calculated for each cell line at the time points shown. The lower images show examples of Con A-FITC signal at the FP and EE regions respectively, as indicated by open and filled arrows respectively.
(TIF)

Figure S4 The presence of CFAS gene attenuates *L. major* dermal infection *in vivo*. BALB/c mice were infected intradermally with 1×10^6^ *L. major* LUC or *L. major* LUC+CFAS and parasites were visualised by bioluminescence imaging, using an IVIS over the course of the infection. Scale bar on left of images shows luminescence activity (photons/second/cm2/sr).
(TIF)

Figure S5 Luciferase activity is downregulated in amastigotes. The luciferase activities of equivalent numbers of *L. major* LUC promastigotes (black bars) and amastigotes (open bars) were compared using an *in vitro* luminescence assay. **** p<10^4^ by unpaired Student’s t-test (n = 3).
(TIF)
