# Supplementary figures and images for: Functional Analysis of Leishmania Cyclopropane Fatty Acid Synthetase
Source: PLoS One. 2012 Dec 10;7(12):e51300. doi: 10.1371/journal.pone.0051300 (PMC3519623; doi:10.1371/journal.pone.0051300)

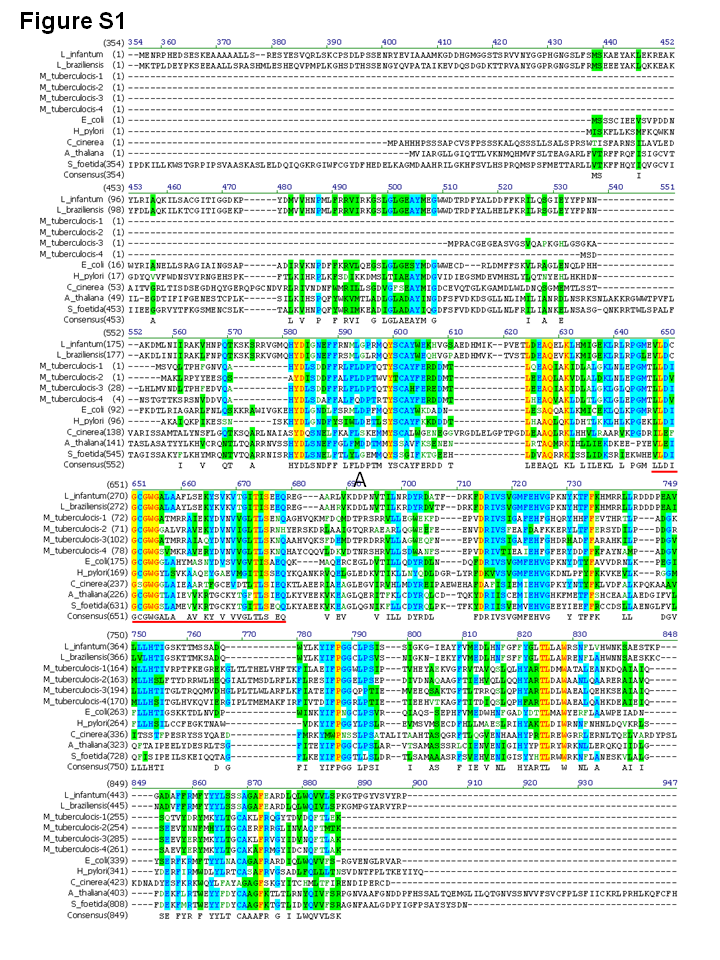

Supplement: Figure S1 — CFAS amino acid sequence analysis. AlignX (Vector NTI tool) was used to align Leishmania CFAS sequences with those from other species. A structurally conserved S-adenosyl-L-methionine (SAM) binding domain is underlined while other highly conserved residues are highlighted. Sequence accession number of the sequences used: XP_001463394 (Leishmania infantum); XP_001562118 (Leishmania braziliensis); NP_334895 (Mycobacterium tuberculosis-1); NP_215159 (Mycobacterium tuberculosis-2); AAC44617 (Mycobacterium tuberculosis-3); NP_215157 (Mycobacterium tuberculosis-4); NP_207214 (Helicobacter pylori); NP_416178 (Escherichia coli); AAL73238 (Coprinopsis cinerea); AAM33848 (Sterculia foetida); NP_188990 (Arabidopsis thaliana). (TIF) [file pone.0051300.s001.tif]

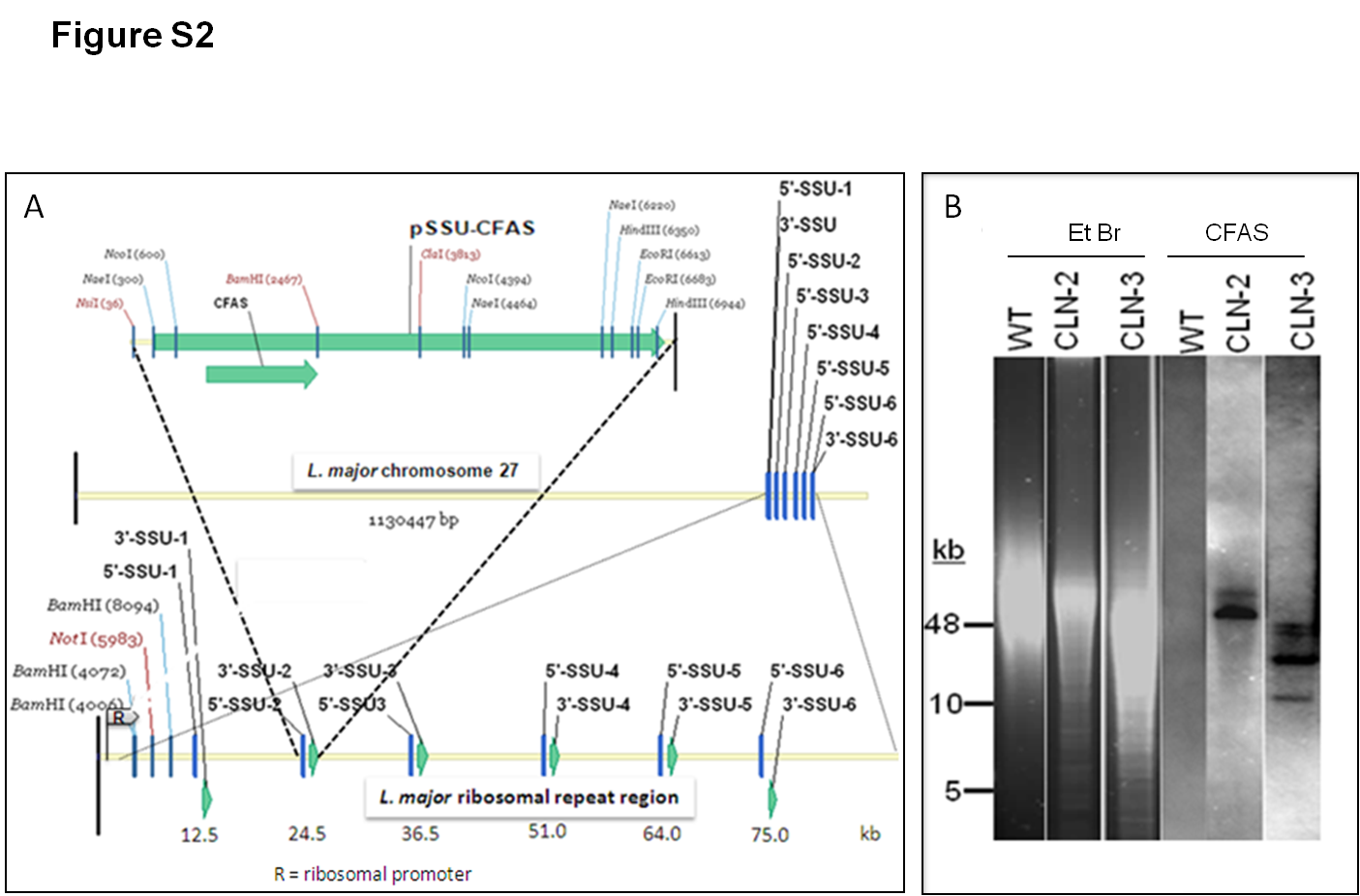

Supplement: Figure S2 — Analysis of CFAS transgene integrations into the ribosomal locus of L. major. (A) Map of the ribosomal integration vector construct (pSSU-NEO-CFAS) and the corresponding region of L. major chromosome 27 with repeated integration sites (SSU) distributed across the locus. The position of the ribosomal promoter (R) is indicated. (B) Southern blot analysis of genomic DNA extracted from wild type (wt) and transgenic L. major mutants expressing CFAS (CLN-2, CLN-3, Table 1). DNA was digested with BamH I, separated by pulsed field gel electrophoresis through 1% agarose, blotted and probed with the CFAS-specific probe shown in Figure 3A. (TIF) [file pone.0051300.s002.tif]

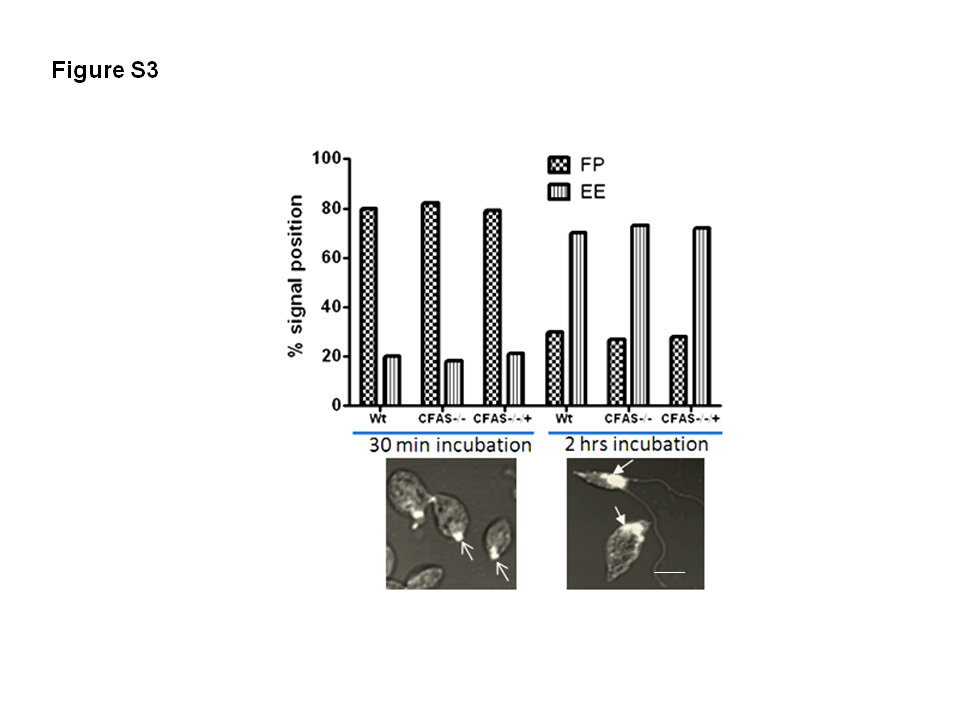

Supplement: Figure S3 — Endocytosis assay. The wild type, null and complemented L. infantum lines used in Figure 6A were incubated with FITC-labeled Con A and uptake stopped after 30 min or 2 hr by fixation with 4% paraformaldehyde. Analysing 100 parasites from each line, the number of parasites with Con A signal at the flagella pocket (FP) or in the early endosomal (EE) regions was counted and the percentage of the total calculated for each cell line at the time points shown. The lower images show examples of Con A-FITC signal at the FP and EE regions respectively, as indicated by open and filled arrows respectively. (TIF) [file pone.0051300.s003.tif]

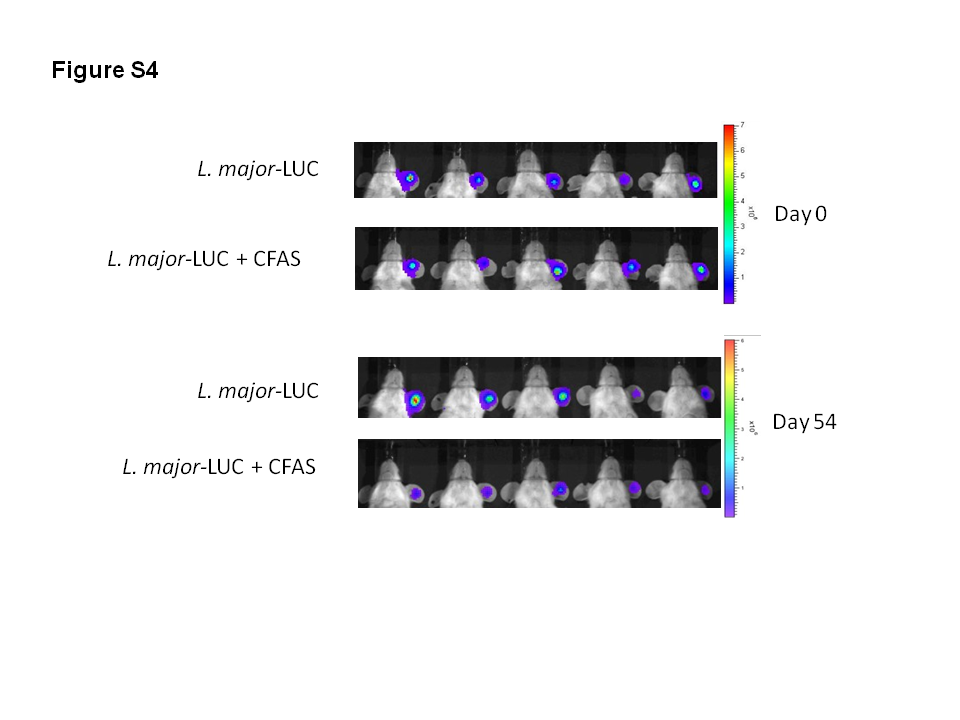

Supplement: Figure S4 — The presence of CFAS gene attenuates L. major dermal infection in vivo. BALB/c mice were infected intradermally with 1×10^6^ L. major LUC or L. major LUC+CFAS and parasites were visualised by bioluminescence imaging, using an IVIS over the course of the infection. Scale bar on left of images shows luminescence activity (photons/second/cm2/sr). (TIF) [file pone.0051300.s004.tif]

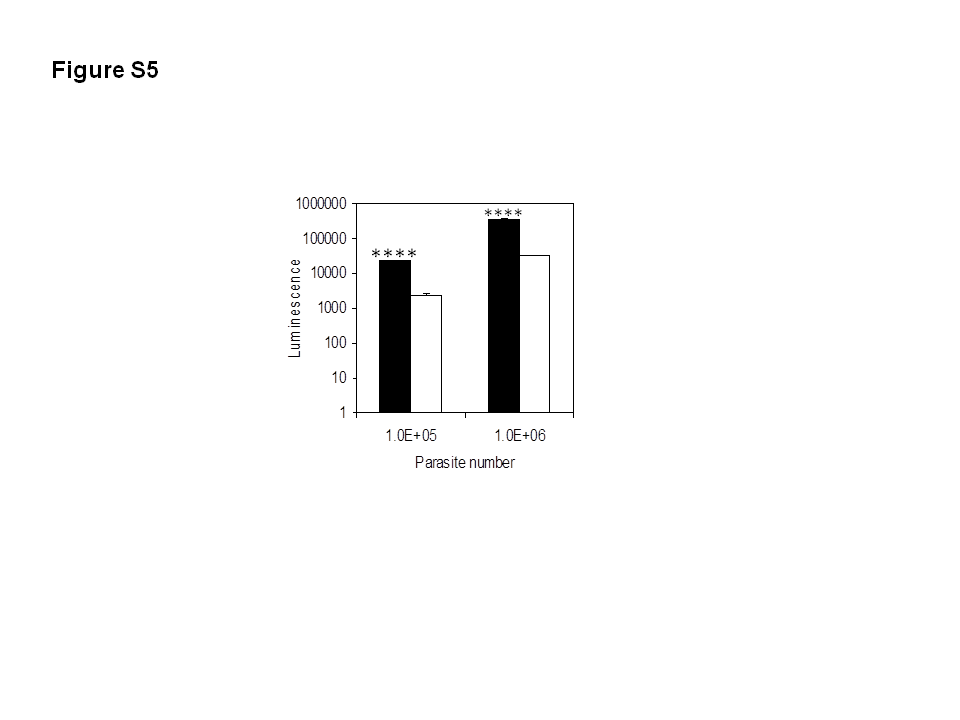

Supplement: Figure S5 — Luciferase activity is downregulated in amastigotes. The luciferase activities of equivalent numbers of L. major LUC promastigotes (black bars) and amastigotes (open bars) were compared using an in vitro luminescence assay. **** p<104 by unpaired Student’s t-test (n = 3). (TIF) [file pone.0051300.s005.tif]
